# Smoking status and voting behaviour and intentions in countries of the former Soviet Union

**DOI:** 10.1038/s41598-025-95632-4

**Published:** 2025-04-24

**Authors:** Andrew Stickley, Yosuke Inoue, Naoki Kondo, Mall Leinsalu, Martin McKee

**Affiliations:** 1 Department of Social Epidemiology, Graduate School of Medicine and School of Public Health, Kyoto University, Kyoto, Japan; 2 Stockholm Centre for Health and Social Change (SCOHOST), Sodertorn University, 141 89 Huddinge, Sweden; 3 Department of Epidemiology and Prevention, Center for Clinical Sciences, National Center for Global Health and Medicine, Tokyo, Japan; 4 Department of Epidemiology and Biostatistics, National Institute for Health Development, Tallinn, Estonia; 5 Department of Health Services Research and Policy, London School of Hygiene & Tropical Medicine, London, UK

**Keywords:** Risk factors, Public health

## Abstract

Smokers experience multiple disadvantages throughout their lives, yet there is another disadvantage, political, that is less widely recognised. Smokers are less likely to vote but only so far in studies conducted in Western democratic regimes. This cross-sectional study aimed to examine the association between current smoking and voting behaviour and intentions in nine countries of the former Soviet Union (FSU). Data were analysed from 18,000 individuals aged ≥ 18 in Armenia, Azerbaijan, Belarus, Georgia, Kazakhstan, Kyrgyzstan, Moldova, Russia and Ukraine, collected in the Health in Times of Transition (HITT) survey in 2010/11. Information was obtained on smoking status and voting behaviour and intentions. In a fully adjusted logistic regression analysis, current smoking was associated with significantly higher odds of ‘never voting’ (not having voted in the past or intending to vote in future) in the pooled sample (OR: 1.29, 95% CI 1.13–1.47). In stratified analyses, smoking was associated with never voting in women but not men and in young but not middle-aged or older adults. The smoking-never voting association was observed in flawed democracies (OR: 1.57, 95% CI 1.07–2.32) and hybrid regimes (OR: 1.31, 95% CI 1.08–1.59) but not in authoritarian regimes (OR: 1.02, 95% CI 0.81–1.29). Smoking is associated with never voting in these FSU countries although not in all population subgroups or types of political regime. A necessary task for future research will be determining the factors associated with not voting among smokers in these countries.

**Supplementary Information:**

The online version contains supplementary material available at 10.1038/s41598-025-95632-4.

## Introduction

The tobacco industry has long promoted smoking in a positive light. Smokers are more attractive, successful, happy, and in control of their lives^1^. Yet the opposite is true. Smoking is associated with disadvantage across the life course^2^. Smokers are more likely to have been brought up in poorer homes as children^3^, have lower educational attainment^4,5^, be less productive at work^6^ with more absenteeism^7^, and experience an increased risk of unemployment in middle-age^8^. Smoking may also be a marker of marital disruption^9^ and has been linked to lower happiness^10^, and increasing social isolation and loneliness in older age^11^. All these are on top of the many consequences for health, including cardiovascular disease^12^, stroke^13^, various cancers^14^, and psychiatric disorders^15,16^, collectively contributing to a higher mortality than among non-smokers^17^.

All of these associations are well-known and form the basis of the case for tobacco control, but there may be others. One is political disadvantage, although the literature is sparser here. Several studies have indicated, for example, that smokers may be less likely to vote than their non-smoking counterparts. An earlier study from Britain found that smokers were 3–4% less likely to vote in the 1979, 1987, and 1997 general elections^18^. Later research undertaken in the American state of Colorado similarly showed that daily smokers had reduced odds of voting in the 2004 November national election^19^, while more recent research among low-income workers in two US cities (Minneapolis and Raleigh) linked current smoking with a reduced likelihood of voting in the last local election^20^. Further support for this association comes from an Irish ecological study in which the prevalence of regular smokers was higher in constituencies where more people abstained^21^. Current smoking has also been associated with the *intention *not to vote in England^22^, while other research found that smokers were also less likely to be registered to vote and actually vote in the 2012 US federal election compared to non-smokers^23^.

Although the factors linking smoking with being less likely to vote are uncertain, various mechanisms might be involved in this process. For example, having a low educational level may be important for the smoking and not voting association, given that previous studies have found that there is a strong educational gradient in smoking behaviour^24^ and that having a low and intermediate level of education increases the probability of being a non-voter^25^. Some research also points to cognitive beliefs as possibly being important for the smoking-voting relationship. In particular, as higher self-efficacy has been linked to both a reduced likelihood of having tried cigarettes^26^ and being more likely to vote in young adults^27^, it can be speculated that reduced levels of self-efficacy might also underlie smoking and not voting in some individuals. It is also possible that the experience of loneliness might be important in this context. Specifically, smoking has been linked longitudinally with increases in loneliness^11^, while recent research that used data from the Netherlands and Germany showed that loneliness is also associated with a reduced probability of having voted^28^. In addition, other research that has examined the association between smoking and not voting has suggested that the potentially poorer health of smokers may play a role in this context^18^ with data from two British birth cohorts showing that poorer health was associated with markedly lower voting especially in young adulthood^29^. It has also been suggested that the stigma associated with smoking might be linked to social marginalisation^20^ and that this may result in a lower propensity to vote.

Building on earlier research undertaken in Western democratic states, the current study will examine the association between smoking and never voting in the countries of the former Soviet Union (FSU). The collapse of the communist system was followed by widespread social and economic turmoil in the FSU countries as witnessed by a sharp decline in Gross Domestic Product (GDP)^30^ (see Appendix 1 in the online supplementary material), a growth in poverty and inequality^31^ as well as catastrophic deterioration in public health with high levels of mortality and decreasing life expectancy. In Russia, for example, male life expectancy at birth fell by 6.6 years in the period from 1989 to 1994, while the corresponding figure for females was 3.3 years^32^. Although there was economic growth and an increase in real incomes in these countries in the 2000s^33,34^, social and economic problems were prolonged in many places, with the poverty rate being over 30% in Armenia and Kyrgyzstan^35,36^ and over 20% in Moldova^37^ in 2010, while life expectancy was still slightly below its 1990 level at this later time point in Belarus, Kazakhstan, Russia and Ukraine^38^. These countries also experienced political instability in the post-Soviet period, with armed conflicts taking place between Armenia and Azerbaijan over Nagorno-Karabakh, in the Transnistria region in Moldova as well as in South Ossetia and Abkhazia in Georgia^31^. Indeed, this study’s authors had to delay data collection in Kyrgyzstan as a result of the violent inter-ethnic conflicts that followed in the wake of the ‘revolution’ and overthrowing of President Bakiyev in 2010^39^. Importantly as regards the current study, in that year, according to the Economist Intelligence Unit’s Democracy Index—which provides a snapshot of the state of democracy in countries across the world through assessments of the electoral process and pluralism, the functioning of government, political participation, political culture and civil liberties—none of our study countries were ranked as full democracies^40^. Rather, they were categorised as ‘flawed democracies’, where elections are free and fair but where there can be problems such as media infringement (Moldova, Ukraine), ‘hybrid regimes’, where elections are marked by widespread irregularities that often prevent them from being free and fair (Kyrgyzstan, Georgia, Russia, Armenia) and ‘authoritarian regimes’, where elections, even if they do occur, are not free and fair and where there is no or very little political pluralism together with widespread censorship and a disregard for civil liberties (Belarus, Kazakhstan, Azerbaijan). Regarding the propensity to vote specifically, this might vary across the regime types given that the political participation index scores (that included voter participation) ranged from a ‘high’ of 6.11/10.00 in Moldova to 3.33 in Belarus, Kazakhstan and Azerbaijan^40^. In this context, as previous research has indicated that voting in these and other countries that are not fully democratic may be affected by a range of factors that are sometimes (e.g. low political trust)^41^, but not always the same (e.g. coercion)^42^ as in democratic societies, it is possible that the smoking-voting association might also differ in this setting.

There is also interesting variation across our study countries in terms of smoking. In 2010 (our study year), the prevalence ranged from 22.5% in Azerbaijan to 33.9% in Belarus, with rates of smoking in the Slavic countries (Belarus, Russia, Ukraine) and Georgia exceeding the average prevalence across the whole of the World Health Organisation’s (WHO) European Region^38^. However, while the prevalence among males (excluding Moldova) ranged from 46.8% in Azerbaijan to 58.7% in Belarus and was above the European average in all of our study countries, the corresponding figures among women were 0.2% (Azerbaijan) and 13.1% (Belarus) and below the WHO European Region average in all of our study countries^38^. Moreover, although smoking rates have declined, especially among men, in some of these countries in recent years^38,43,44^, as can be seen from the above-mentioned figures, smoking remains common^45^ and has been linked to both social and economic disadvantage^46,47^. Determining whether smoking is also linked to political disadvantage is an important task, given that the association between smoking and voting has only been examined in Western democracies until now.

Beyond the factors outlined above, these countries also provide an interesting setting to examine the association between smoking and not voting, given that there is evidence that the decline in voter turnout in the post-communist countries in Europe has been sharper than in the established European democracies in the post-1990 period^48^, although there is some indication that the situation may be more variable in our study countries^49^. Despite this, as yet, comparatively little is known about the factors affecting voter turnout in the FSU countries. This is an important omission. In contrast to the Soviet period, where voting was all but mandatory and where not voting was regarded as an expression of political dissatisfaction and a political act rather than an expression of apathy^50^, some research has indicated that there may be a variety of reasons for not voting in these countries in the post-Soviet period^41,51^ and that the sociodemographic factors associated with not voting may also vary across these countries^52^. Given this, it is also possible that the association between smoking and abstention may differ across different groups and types of political regime in this study setting.

Thus, this cross-sectional study has three main aims: (i) to determine whether there is an association between smoking status and voting in the FSU countries; (ii) to explore whether the associations vary by sex and/or age or in relation to other sociodemographic factors; and (iii) to examine if these associations vary among different types of regime. Based on previous research, it is hypothesised that smoking will be associated with not voting in these countries. However, given the absence of prior research on sociodemographic differences in the smoking and not voting association, no hypotheses regarding these potential associations will be proposed.

## Methods

### Study participants

Data came from the Health in Times of Transition (HITT) survey in nine FSU countries (Armenia, Azerbaijan, Belarus, Georgia, Kazakhstan, Kyrgyzstan, Moldova, Russia, and Ukraine) in 2010–2011. Multi-stage random sampling with stratification by region and settlement type (urban/rural) was undertaken in each country to obtain nationally representative samples. Within the primary sampling units (approximately 100–200 per country), random route procedures were used to select households, and one individual aged 18 or above was then randomly selected to participate in the survey (using the nearest birthday). Information was obtained during face-to-face interviews undertaken by trained interviewers using a standard questionnaire. Interviewees could respond in either their own country’s language or Russian, except for in Russia and Belarus, where only Russian was used. Exclusion criteria included being homeless, in the military, incarcerated, institutionalised, hospitalised, or intoxicated at the time of the survey. Across the nine countries, data were obtained from 18,000 respondents. In six countries, the sample size was 1800 persons. Larger samples were obtained in Russia and Ukraine (3000 and 2000 persons, respectively) to reflect these countries’ larger size and more regionally diverse populations. Georgia also had a larger sample size (2200 persons) after a 400-person booster survey was undertaken at the end of 2010 to ensure the sample was more representative. Survey response rates ranged from 47% (Kazakhstan) to 83% (Georgia) across the study countries^53^. Further details of the HITT project are available elsewhere^54^. The ethics committee at the London School of Hygiene and Tropical Medicine provided permission for the survey, which was undertaken in accordance with the 1964 Helsinki Declaration. All respondents provided informed consent.

### Measures

#### Dependent and independent variables

Regarding their *voting behaviour and intentions*, respondents were asked to select one of five response options: (i) Did it and will do it in future (i.e., ‘always voting’); (ii) Did it but will not do it in future (i.e., ‘past voting only’); (iii) I did not participate, but I will participate in future (i.e., ‘future voting only’); (iv) I did not participate and I will not participate in the future (i.e., ‘never voting’); (v) Don’t know. In line with a previous study that examined the association between smoking status and intending not to vote^22^, our focus was on non-participation in this study. Given this, the response option (i) always voting was used as the reference category, while option (iv) never voting was used as the outcome. Those who used the other response options were removed from the analysis. In addition, our focus on option (iv) was also guided by other factors: we wished to focus on those whose future intentions coincided with their past behaviours because previous research has indicated that while there can sometimes be a disparity between individuals’ expressed intention to vote in the future and their actual future voting behaviour, few of those who state that they will not vote in future actually vote^55^. *Smoking status* was assessed using a three-category variable that differentiated never smokers (reference category) from former smokers and current smokers (those who reported smoking at least one cigarette per day).

#### Covariates

Information was collected on the sex (male, female) and age of the respondents, subsequently categorised as 18–34, 35–59 and ≥ 60. Education was assessed as low (incomplete secondary education or below), mid (completed secondary/secondary special education), or high (completed/non-finished higher education). For marital status, respondents were categorised as being married/cohabiting, never married, or divorced/widowed; for location, they were categorised as living in either urban or rural areas. Four categories were used to classify occupation: (i) those in regular/irregular paid work (comprising people in regular/irregular paid work, individual entrepreneurs, the self-employed, full-time working students); (ii) non-working individuals (homemakers, those on paternity leave, full-time non-working students, the retired); (iii) farmers/agricultural labourers (peasants); (iv) unemployed persons. The financial status of each respondent’s household was assessed as good/very good, average, or bad/very bad. Respondents rated their own health using three categories, good/very good, fair, or poor/very poor. Problem drinking was assessed with the CAGE questionnaire^56^ with a score ≥ 2 being used to categorise cases^57^. Finally, as previous research has indicated that political trust/distrust is linked to both smoking^58^ and voting behaviour^59–61^ a measure of political distrust was also included in the analysis. This was assessed with the question, “Please tell me on a score of 1–10, where 1 means complete distrust and 10—absolute trust, how much you personally trust each of the following institutions:” (i) president of the country; (ii) government; (iii) parliament; (iv) county/regional council; (v) mayoralty; (vi) political parties. After reverse-coding and summing the scores for the 6 items, we calculated their mean where higher values indicated greater political distrust. Cronbach’s alpha for the scale was 0.93.

#### Statistical analysis

Multiple imputation was used to generate 20 datasets to account for the variables with missing data. More specifically, the chained equation method was employed with linear, logistic, ordered logistic and multinomial logistic regression models used for continuous, binary, discrete or categorical variables, respectively. Rubin’s rules were then followed to combine imputation estimates. We first calculated descriptive statistics stratified by smoking status. Next, as we were examining a binary outcome (never voting vs. always voting) and in line with the previous studies that have examined the relationship between smoking status and not voting^19,22,23^, logistic regression was used to examine the association between smoking status and voting behaviour in the pooled sample. We tested five models. Model 1 examined the unadjusted association between smoking and never voting, while Model 2 added sociodemographic variables—sex, age, education, marital status, occupation, household finances and location. Model 3 further included self-rated health, while problem drinking was added in Model 4. The fully adjusted Model 5 included the same variables as in Model 4, with the addition of political distrust. To check the analysis for the possibility of multicollinearity, variance inflation factors (VIFs) were calculated. It has been suggested that a value higher than 5 to 10 suggests multicollinearity^62^. In this case, the highest VIF value was 4.33 (for political distrust). Sex- and age-stratified analyses and analyses stratified by education, household financial situation and self-rated health were then undertaken using the same model building process outlined above to determine whether these factors might affect the smoking and never voting association. Finally, we examined whether the association between smoking status and voting behaviour differed according to the type of political regime, while using the same model-building process.

All analyses were performed with the statistical package STATA version 17.0 (Stata Corp LP, College Station, Texas). All the pooled analyses were adjusted for country with the use of dummy variables^63,64^. The results are presented as odds ratios (OR) and 95% confidence intervals (CI). The level of statistical significance was *p* < .05 (two-tailed).

## Results

Most respondents were never smokers (64.1%), 10.0% were former smokers, and one-quarter (25.9%) were current smokers. Over three-quarters of the sample were ‘always voters’ (76.1%), whereas 11.1% were ‘never voters’. The imputed sample characteristics stratified by smoking status are presented in Table 1 and stratified by voting behaviour and intentions in Appendix 2 in the online supplementary information. The prevalence of never voting was higher among women, those aged 18–34, the lower educated, the never married, those with bad household finances, people living in urban areas and authoritarian regimes.

**Table 1:** Characteristics of the study sample after multiple imputation.

	Total (*N* = 18000)	Smoking status		
		Never smoker (64.1%)	Former smoker (10.0%)	Current smoker (25.9%)
Sex				
Men	43.5%	23.8%	71.2%	81.5%
Women	56.5%	76.2%	28.8%	18.5%
Age				
18–34	37.8%	36.2%	35.1%	42.6%
35–59	43.3%	42.0%	40.8%	47.6%
≥ 60	18.9%	21.8%	24.1%	9.8%
Education				
High	27.5%	27.5%	32.1%	25.8%
Mid	59.4%	58.1%	57.4%	63.6%
Low	13.1%	14.5%	10.5%	10.6%
Marital status				
Never married	20.6%	19.7%	19.1%	23.1%
Married/cohabiting	62.1%	60.4%	66.0%	64.8%
Divorced/widowed	17.4%	19.9%	14.9%	12.0%
Occupation				
Non-working	36.6%	44.7%	33.3%	17.8%
Regular/irregular paid work	47.4%	40.3%	54.7%	62.2%
Farmers/agricultural labourers	4.2%	3.9%	3.5%	5.2%
Unemployed	11.8%	11.2%	8.5%	14.7%
Household finances				
Good/very good	22.4%	22.7%	22.5%	21.6%
Average	57.3%	56.7%	58.5%	58.3%
Bad/very bad	20.3%	20.7%	19.0%	20.1%
Location				
Urban	60.4%	57.8%	65.5%	64.3%
Rural	39.6%	42.2%	34.5%	35.7%
Self-rated health				
Good/very good	40.6%	39.5%	37.1%	44.8%
Fair	40.9%	39.5%	43.8%	43.3%
Poor/very poor	18.4%	21.0%	19.0%	12.0%
Problem drinking	13.7%	5.3%	23.3%	30.8%
Political distrust, mean	4.7	4.5	5.1	4.9

In an unadjusted logistic regression analysis, being a former smoker was not associated with never voting in the total sample, whereas current smokers had 21% higher odds for never voting (OR: 1.21, 95% CI 1.08–1.35) (Table 2, Model 1). Including the covariates in Models 2–5 had little effect, so being a current smoker continued to be significantly associated with never voting in the fully adjusted Model 5 (OR: 1.29, 95% CI 1.13–1.47). Other variables associated with never voting were female sex, younger age, having less education, not being married, not working, urban residence, good self-rated health and greater political distrust.

**Table 2:** Association between smoking status and never voting (not having voted in the past and planning not to vote in the future) in the countries of the former Soviet Union (*N* = 15631).

	Model 1	Model 2	Model 3	Model 4	Model 5
	OR (95% CI)	OR (95% CI)	OR (95% CI)	OR (95% CI)	OR (95% CI)
Smoking status					
Never smoker	Ref.	Ref.	Ref.	Ref.	Ref.
Former smoker	0.99 (0.83–1.18)	1.10 (0.91–1.33)	1.10 (0.92–1.33)	1.09 (0.90–1.32)	0.99 (0.82–1.18)
Current smoker	1.21 (1.08–1.35) **	1.28 (1.12–1.46)***	1.29 (1.13–1.47)***	1.26 (1.10–1.45)**	1.29 (1.13–1.47)***
Sex (Woman)		1.10 (0.97–1.24)	1.10 (0.98–1.25)	1.12 (0.99–1.27)	1.13 (1.00–1.28)*
Age					
18–34		Ref.	Ref.	Ref.	Ref.
35–59		0.77 (0.68–0.88)***	0.78 (0.69–0.89)***	0.78 (0.69–0.89)***	0.85 (0.74–0.96)**
≥60		0.48 (0.39–0.58)***	0.48 (0.39–0.58)***	0.48 (0.39–0.58)***	0.53 (0.44–0.65)***
Education					
High		Ref.	Ref.	Ref.	Ref.
Mid		1.26 (1.11–1.42)***	1.25 (1.11–1.42)***	1.25 (1.11–1.42)***	1.39 (1.23–1.56)***
Low		1.57 (1.31–1.89)***	1.55 (1.29–1.87)***	1.55 (1.29–1.87)***	1.46 (1.22–1.74)***
Marital status					
Married/cohabiting		Ref.	Ref.	Ref.	Ref.
Never married		1.26 (1.10–1.45)**	1.25 (1.09–1.44)**	1.25 (1.09–1.44)**	1.32 (1.15–1.51)***
Divorced/widowed		1.34 (1.15–1.55)***	1.33 (1.15–1.54)***	1.33 (1.15–1.54)***	1.25 (1.08–1.44)**
Occupation					
Non-working		Ref.	Ref.	Ref.	Ref.
Regular/irregular paid work		0.75 (0.66–0.86)***	0.76 (0.67–0.86)***	0.76 (0.67–0.86)***	0.74 (0.65–0.84)***
Farmers/agricultural labourers		0.54 (0.40–0.75)***	0.55 (0.40–0.75)***	0.55 (0.40–0.75)***	0.56 (0.41–0.76)***
Unemployed		0.89 (0.75–1.06)	0.90 (0.76–1.07)	0.90 (0.76–1.07)	0.91 (0.77–1.07)
Household finances					
Good/very good		Ref.	Ref.	Ref.	Ref.
Average		1.02 (0.90–1.16)	1.03 (0.91–1.17)	1.03 (0.90–1.17)	1.04 (0.92–1.18)
Bad/very bad		1.37 (1.16–1.61)***	1.36 (1.15–1.61)***	1.35 (1.14–1.60)***	1.24 (1.05–1.46)*
Location (rural)		0.74 (0.66–0.82)***	0.73 (0.66–0.82)***	0.73 (0.66–0.82)***	0.67 (0.60–0.75)***
Self-rated health					
Good/very good			Ref.	Ref.	Ref.
Fair			0.92 (0.82–1.04)	0.92 (0.81–1.03)	0.84 (0.74–0.94)**
Poor/very poor			1.05 (0.89–1.25)	1.05 (0.89–1.25)	0.91 (0.77–1.08)
Problem drinking				1.11 (0.96–1.30)	1.02 (0.88–1.19)
Political distrust					1.05 (1.03–1.07)***

OR, Odds ratio; CI, Confidence interval; Ref, Reference category.

*** *p* < .001, ** *p* < .01, * *p* < .05.

All models were adjusted for country. Sample size varied between 15,631 and 15,646.

In a sex-stratified analysis, there was no association between former or current smoking and never voting among men (Table 3). In contrast, in the fully adjusted Model 5, current smoking among women was associated with 59% higher odds of never voting (OR: 1.59, 95% CI 1.27–1.99). In the age-stratified analysis, current smoking was not associated with never voting in adults aged 35–59 or those aged 60 and above in any of the models. However, among young adults (age 18–34), current smoking was significantly associated with never voting in all of the models, with 29% higher odds in the fully adjusted Model 5 (OR: 1.29, 95% CI 1.06–1.57).

**Table 3:** Sex- and age-specific associations between smoking status and never voting (not having voted in the past and planning not to vote in the future) in the countries of the former Soviet Union.

	Model 1	Model 2	Model 3	Model 4	Model 5
	OR (95% CI)	OR (95% CI)	OR (95% CI)	OR (95% CI)	OR (95% CI)
Sex					
Men (*N* = 6719)					
Smoking status					
Never smoker	Ref.	Ref.	Ref.	Ref.	Ref.
Former smoker	0.83 (0.65–1.06)	0.91 (0.71–1.17)	0.90 (0.70–1.15)	0.90 (0.70–1.15)	0.89 (0.69–1.14)
Current smoker	1.11 (0.94–1.31)	1.06 (0.89–1.26)	1.05 (0.89–1.25)	1.05 (0.88–1.25)	1.03 (0.86–1.23)
Women (*N* = 8902)					
Smoking status					
Never smoker	Ref.	Ref.	Ref.	Ref.	Ref.
Former smoker	1.47 (1.11–1.96)**	1.43 (1.07–1.91)*	1.43 (1.07–1.92)*	1.40 (1.04–1.88)*	1.29 (0.96–1.73)
Current smoker	1.89 (1.54–2.33)***	1.74 (1.40–2.16)***	1.75 (1.41–2.17)***	1.70 (1.36–2.12)***	1.59 (1.27–1.99)***
Age					
18–34 (*N* = 5643)					
Smoking status					
Never smoker	Ref.	Ref.	Ref.	Ref.	Ref.
Former smoker	1.12 (0.85–1.47)	1.15 (0.87–1.53)	1.16 (0.87–1.54)	1.14 (0.85–1.51)	1.10 (0.83–1.47)
Current smoker	1.26 (1.07–1.49)**	1.34 (1.10–1.62)**	1.34 (1.11–1.63)**	1.31 (1.08–1.60)**	1.29 (1.06–1.57)*
35–59 (*N* = 6939)					
Smoking status					
Never smoker	Ref.	Ref.	Ref.	Ref.	Ref.
Former smoker	1.03 (0.78–1.35)	1.07 (0.80–1.44)	1.08 (0.80–1.44)	1.07 (0.79–1.43)	1.02 (0.76–1.37)
Current smoker	1.14 (0.96–1.34)	1.17 (0.94–1.44)	1.17 (0.95–1.45)	1.15 (0.93–1.43)	1.10 (0.89–1.37)
≥ 60 (*N* = 3039)					
Smoking status					
Never smoker	Ref.	Ref.	Ref.	Ref.	Ref.
Former smoker	0.67 (0.44–1.02)	0.99 (0.60–1.63)	0.98 (0.59–1.63)	0.98 (0.59–1.63)	0.93 (0.56–1.55)
Current smoker	0.79 (0.54–1.16)	1.18 (0.73–1.90)	1.19 (0.74–1.92)	1.19 (0.74–1.93)	1.17 (0.72–1.90)

Model 1 examined the unadjusted association between smoking status and never voting; Model 2 additionally adjusted for sex, age (where appropriate), education, marital status, household finances, occupation and location; Model 3 was additionally adjusted for self-rated health; Model 4 was additionally adjusted for problem drinking; Model 5 was additionally adjusted for political distrust.

All models were adjusted for country. Sample size varied between 6719 and 6730 for men, 8902 and 8920 for women, 5643 and 5665 for individuals aged 18–34 years old, 6939 and 6949 for individuals aged 35–59 years old, and 3039 and 3049 for individuals aged ≥ 60 years old.

OR, Odds ratio; CI, Confidence interval; Ref, Reference category.

*** *p* < .001, ** *p* < .01, * *p* < .05.

When the analysis was stratified by education, in the fully adjusted Model 5, there was no association between smoking status and never voting among the high educated (Appendix 3 in the online supplementary information). In contrast, current smoking was associated with higher odds of never voting in individuals with a mid (OR: 1.20, 95% CI 1.00–1.43) and low education (OR: 1.56, 95% CI 1.01–2.41). In the household finances-stratified analysis, in Model 5, current smoking was associated with higher odds of never voting in both those with good (OR: 1.38, 95% CI 1.04–1.83) and bad household finances (OR: 1.40, 95% CI 1.01–1.93) (Appendix 4 in the online supplementary information). Finally, in an analysis stratified by self-rated health status, current smoking was associated with never voting only among those with fair self-rated health (OR: 1.34, 95% CI 1.08–1.67) (Appendix 5 in the online supplementary information).

Turning to regime type, there was no association between former or current smoking and never voting in authoritarian regimes (Table 4). However, after full adjustment, current smoking was associated with significantly higher odds of never voting in flawed democracies (OR: 1.57, 95% CI 1.07–2.32) and hybrid regimes (OR: 1.31, 95% CI 1.08–1.59).

**Table 4:** Regime-specific associations between smoking status and never voting (not having voted in the past and planning not to vote in the future) in the countries of the former Soviet Union.

	Model 1	Model 2	Model 3	Model 4	Model 5
	OR (95% CI)	OR (95% CI)	OR (95% CI)	OR (95% CI)	OR (95% CI)
Political regime					
Flawed (*N* = 3393)					
Smoking status					
Never smoker	Ref.	Ref.	Ref.	Ref.	Ref.
Former smoker	1.19 (0.77–1.84)	1.18 (0.74–1.87)	1.17 (0.74–1.86)	1.23 (0.77–1.96)	1.21 (0.76–1.93)
Current smoker	1.66 (1.21–2.27)**	1.44 (0.98–2.10)	1.44 (0.98–2.10)	1.60 (1.09–2.37)*	1.57 (1.07–2.32)*
Hybrid (*N* = 7526)					
Smoking status					
Never smoker	Ref.	Ref.	Ref.	Ref.	Ref.
Former smoker	0.86 (0.67–1.11)	0.97 (0.73–1.27)	0.96 (0.73–1.27)	0.94 (0.71–1.24)	0.89 (0.67–1.18)
Current smoker	1.35 (1.16–1.58)***	1.41 (1.17–1.70)***	1.41 (1.17–1.70)***	1.36 (1.13–1.65)**	1.31 (1.08–1.59)**
Authoritarian (*N* = 4700)					
Smoking status					
Never smoker	Ref.	Ref.	Ref.	Ref.	Ref.
Former smoker	1.15 (0.86–1.53)	1.29 (0.95–1.75)	1.30 (0.96–1.76)	1.28 (0.94–1.74)	1.23 (0.90–1.67)
Current smoker	0.92 (0.77–1.11)	1.07 (0.86–1.34)	1.08 (0.86–1.35)	1.05 (0.84–1.33)	1.02 (0.81–1.29)

Model 1 examined the unadjusted association between smoking status and never voting; Model 2 additionally adjusted for sex, age, education, marital status, occupation, household finances and location; Model 3 was additionally adjusted for self-rated health; Model 4 was additionally adjusted for problem drinking; Model 5 was additionally adjusted for political distrust.

Sample size varied between 3393 and 3401 for flawed regimes, 7526 and 7544 for hybrid regimes, 4700 and 4711 for authoritarian regimes.

OR: Odds ratio; CI: Confidence interval; Ref: Reference category.

*** *p* < .001, ** *p* < .01, * *p* < .05.

## Discussion

This study is a reminder that there is a political dimension to tobacco control^65,66^. Taking advantage of an especially rich dataset that includes 18,000 adults in nine countries with different political regimes, it adds new insights to the sparse literature on the association between smoking status and voting behaviour. A pooled logistic regression analysis showed that current smokers were significantly more likely to be in the ‘never voting’ category (i.e. not having voted in the past or intending to vote in future) than those who had never smoked, but after disaggregation, this was only true for women and young adults. Other stratified analyses showed that a relationship between smoking and never voting was also observed in groups where there is usually a higher propensity to vote in some countries, such as those in a better financial position^67^. Current smoking was also linked to never voting in flawed democracies and hybrid regimes but not authoritarian regimes.

The finding that current smoking was associated with never voting in the total sample is consistent with previous studies from Britain^18^ and the US^19,20^ as well as with other research from the US, which showed that daily smoking was associated with being less likely to be registered to vote and actually voting^23^, and a study that linked current smoking with intending not to vote in England^22^. Although the factors linking smoking with a lower likelihood of voting are unclear several mechanisms have been proposed. For example, it has been suggested that smoking may be a marker of bad health that could affect one’s ability to vote^18^ and that having low trust in people may mediate the association between smoking and registering to vote^23^. Alternatively, it is also possible that time preferences might play a role in this association. Specifically, research has shown that smokers are more impatient, with a high time discount rate^68,69^, have a present time bias/are more impulsive^70^, while other studies have linked patience and impatience/present bias, respectively, to voting and not voting^71–73^. This might be relevant for the current study given that recent research from Russia has also found that smoking is associated with a higher individual discount and time preference rate^74,75^.

In the sex-stratified analysis, women who smoked had significantly higher odds for never voting whereas male smoking and voting were not linked. To the best of our knowledge, until now there has been no research examining if there are sex or age differences in the association between smoking and not voting so the potential mechanisms underlying this association can only be speculated on. In the Soviet Union smoking was regarded as a ‘male habit’ and female smoking was frowned upon^76^. However, the collapse of the communist system coincided with a rapid growth in women’s smoking, driven by the intensive targeting of them by transnational tobacco companies, including introducing new lighter brands and brands aimed explicitly at women^77^. The growth in smoking was especially marked in young women living in urban areas, where it was thought to be a sign of emancipation and social ascension^78^. Indeed, one study found that women who smoke in the FSU countries are more likely to have non-traditional, anti-communist leanings and suggested that smoking might be a way of asserting their individuality^79^. In this context it can be speculated that not voting might also be a way of asserting individuality given that there was strong socio-political pressure to vote during the Soviet period^80^. Alternatively, other evidence suggests that the social and economic upheaval of the post-Soviet period may have impacted especially negatively on women^81^, with continuing inequalities seen in various spheres—including political empowerment. In particular, according to the Global Gender Gap Report, which measures gender inequality, in every one of our study countries (excluding Belarus where there was no information available), the political empowerment ranking (based on the number of women in parliament, in ministerial positions and years with a female head of state) was below the overall country ranking in 2010 and ranged from rank 65 (Kyrgyzstan) to rank 119 (Georgia) out of 134 ranked countries, with four of our study countries having political empowerment rankings below 100 (Ukraine, Armenia, Azerbaijan, Georgia)^82^. This may be relevant given that the greater representation of women in national parliaments has been linked to their increased interest in politics^83^, while other research has linked a lack of interest in politics to not voting^25^. At the same time, women’s political underrepresentation might also result in specific issues, such as violence against women, being less likely to receive attention from legislators^84^, which might be important for our examined association given that violence against women is common in the FSU countries^81^, while being a victim of intimate partner violence has been linked to an increased risk of smoking^85^.

The ‘double burden’ of many post-Soviet women i.e. taking care of the home while having paid employment^86^ might also connect smoking with never voting. Specifically, it gives rise to both high levels of stress^87^ to which smoking might be one response^46^ that is used to calm nerves^88^, and also ‘time poverty’ (a lack of free or discretionary time)^89^ which may impede a women’s ability to vote. Support for this latter supposition comes from an earlier study in Russia where the main reason for not voting across three election cycles in 1995–2003 was “[I] could not get to the polling station on the day”^41^. Poorer mental health may also link these phenomena as previous research undertaken in Belarus, Kazakhstan, Russia and Ukraine found that psychological distress was associated with smoking in women but not men^86^, while psychological distress has also been associated with not voting among women in these countries^90^.

Young adult smokers had increased odds for never voting when compared with their middle-aged and older counterparts. There is extensive evidence from many countries that younger adults are less likely to vote^91^, something that has also been observed in our study countries^92,93^. This could reflect a lower ability to vote, with young people being more mobile, more likely to be living in temporary accommodation, and separated from their communities^94^. This may be relevant to our findings as transitional lifestyles may also increase susceptibility for smoking initiation in younger adults^95^. Other factors might also be important however. For example, there is some indication that young adults may have been disproportionately affected by unemployment in these countries during the political transition^96,97^, while other research has linked unemployment to a higher risk of both not voting^98^ and smoking^99^ in this age range (although it should be noted that occupational status was controlled for in our analyses).

In further stratified analyses, current smoking was associated with not voting among those with a mid and low level of education, individuals whose household finances were good or bad and who reported that their self-rated health was fair. Previous research in Western countries has suggested that socioeconomic disadvantage such as having a lower level of education^25^, lower income^100^, and being in poorer health^18^ are associated with an increased likelihood of not voting, while an earlier study showed that there may be similar associations in the FSU countries as low education and a worse economic position were both associated with an increased probability of being a non-voter in Belarus and Russia, while low education but not economic position was linked to non-voting in Ukraine^52^. Thus, our finding that smoking may be associated with never voting in both the low educated and those who are economically deprived is in line with earlier research linking socioeconomic disadvantage and abstention. In contrast, the fact that smokers who were in a better financial position were also less likely to vote in our study countries indicates that more research is needed on whether specific sociodemographic factors are important for the association between smoking and not voting and on the specific mechanisms that are relevant for these associations in both our study countries and other locations.

Given that previous studies on the smoking and not voting association were single country studies undertaken in Western democratic countries, perhaps the most important new finding was that current smoking was linked to never voting in flawed democracies and hybrid regimes (which come closest to the systems in which previous research was conducted) but not in authoritarian regimes. Unlike in settings where elections are free and fair but with problems (flawed democracies) or often not free and fair (hybrid regimes), those in authoritarian regimes are never free and fair^40^. Of relevance is a recent study that reported how coercion of opposition figures and fear of reprisals was positively associated with voter turnout in authoritarian regimes^42^ while other research has also highlighted the role that voter intimidation can play in such regimes^101^. Thus, it is possible that these types of factors might have affected the smoking-voting association we observed in our authoritarian regimes. This is supported by a recent study reporting forced or ‘encouraged’ voting in both Belarus and Azerbaijan^102^.

This study has several limitations. First, as all the information was obtained from self-reports it is possible that social desirability bias may have been an issue, especially as voting is often considered a sensitive behaviour that has sometimes been over-reported in previous studies^103^. Second, it is possible that potentially important variables were not included in the analysis. For example, prior research has shown that both smoking^104^ and voting behaviour^105^ might be transmitted intergenerationally. Third, voting behaviours and intentions were not reported in relation to different types of election (i.e. national or local). To better specify the smoking-voting association future studies should examine whether the type of election is important for this association. Finally, this study used data from 2010/11. Since that time several of these countries have changed their regime categorisation (e.g. Russia and Kyrgyzstan were authoritarian regimes in the 2022 Democracy Index)^106^ and future research is thus needed to determine whether this might have affected the associations observed in this study.

In conclusion, this study showed that smoking is associated with voting behaviour in the FSU countries although this association is not observed in all population subgroups or types of political regime. Importantly, the results of this study highlight that the relationship between smoking and electoral disadvantage seemingly extends beyond Western democracies and is seen in other types of electoral regime (although not in authoritarian regimes). An important task for future research will thus be to examine if the association between smoking and not voting is observed in other non-fully democratic regimes in other parts of the world and determine what factors are associated with not voting among smokers in these populations.

## Electronic supplementary material

Below is the link to the electronic supplementary material.

Supplementary Material 1

## Data Availability

The data used in this study are available from the first author (amstick66@gmail.com) upon reasonable request.
