# Supplementary material for: Smoking status and voting behaviour and intentions in countries of the former Soviet Union
Source: Sci Rep. 2025 Apr 24;15:14308. doi: 10.1038/s41598-025-95632-4 (PMC12022174; doi:10.1038/s41598-025-95632-4)
Supplement: Supplementary file 1 — Supplementary Material 1 [file 41598_2025_95632_MOESM1_ESM.pdf]

# Supplementary information

Smoking status and voting behaviour and intentions in countries of the former Soviet Union

Andrew Stickley, Yosuke Inoue, Naoki Kondo, Mall Leinsalu, Martin McKee

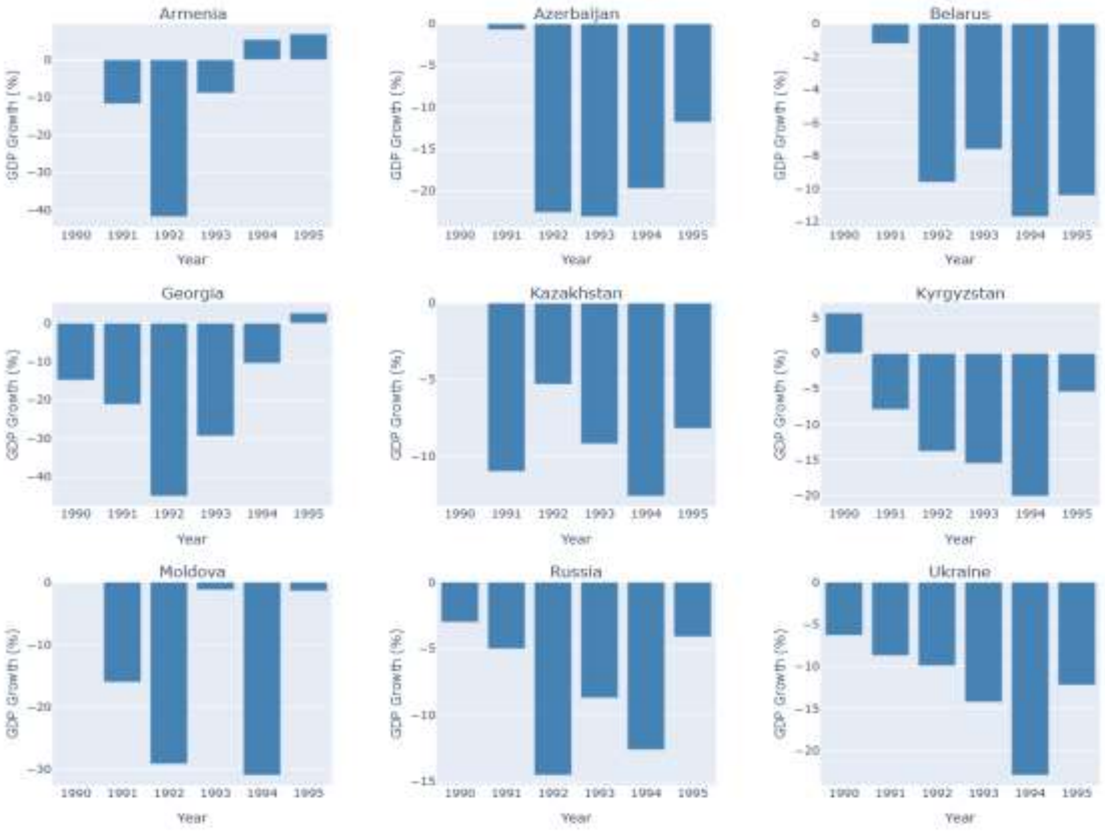


**Appendix 1** Gross Domestic Product (GDP) Growth (annual %) in the study countries in 1990-1995

Source: World Bank Open Data. Available at: https://data.worldbank.org/ (Accessed 19 March 2025)

**Appendix 2** Distribution of voting behaviours in the countries of the former Soviet Union (N = 18000)

| | Voting behaviour | | | |
|---|---|---|---|---|
| | Always voting | Future voting only | Past voting only | Never voting |
| All participants | 76.1% | 7.7% | 5.2% | 11.1% |
| Sex | | | | |
| Male | 75.6% | 7.7% | 6.0% | 10.6% |
| Female | 76.4% | 7.6% | 4.5% | 11.5% |
| Age | | | | |
| 18-34 | 70.5% | 7.0% | 9.6% | 12.8% |
| 35-59 | 78.8% | 7.8% | 2.9% | 10.6% |
| ≥ 60 | 81.0% | 8.7% | 1.5% | 8.8% |
| Education | | | | |
| High | 78.2% | 7.5% | 4.7% | 9.6% |
| Mid | 75.0% | 7.8% | 5.4% | 11.8% |
| Low | 76.3% | 7.6% | 4.9% | 11.2% |
| Occupation | | | | |
| Non-working | 74.8% | 7.6% | 5.9% | 11.7% |
| Regular/irregular paid work | 77.7% | 7.7% | 4.1% | 10.5% |
| Farmers/agricultural labourers | 81.7% | 5.6% | 5.8% | 6.8% |
| Unemployed | 71.7% | 8.4% | 7.0% | 12.9% |
| Marital status | | | | |
| Never married | 66.5% | 7.3% | 12.6% | 13.6% |
| Married/cohabiting | 79.0% | 7.3% | 3.6% | 10.1% |
| Divorced/widowed | 76.9% | 9.4% | 2.0% | 11.8% |
| Household finances | | | | |
| Good/very good | 74.6% | 7.5% | 7.3% | 10.6% |
| Average | 77.3% | 7.0% | 4.9% | 10.8% |
| Bad/very bad | 74.3% | 9.6% | 3.5% | 12.5% |
| Location | | | | |
| Urban | 73.4% | 8.4% | 5.8% | 12.4% |
| Rural | 80.2% | 6.5% | 4.3% | 9.1% |
| Self-rated health | | | | |
| Good/very good | 72.5% | 7.4% | 8.1% | 12.0% |
| Fair | 78.6% | 7.6% | 3.6% | 10.2% |
| Poor/very poor | 78.5% | 8.4% | 2.1% | 11.0% |
| Problem drinking | | | | |
| Yes | 76.3% | 7.5% | 5.2% | 11.0% |
| No | 74.6% | 8.8% | 5.2% | 11.5% |
| Political Regime | | | | |
| Flawed | 84.0% | 6.6% | 3.7% | 5.7% |
| Hybrid | 75.0% | 9.1% | 4.9% | 11.1% |
| Authoritarian | 72.3% | 6.1% | 6.7% | 14.9% |

**Appendix 3** Education-specific associations between smoking status and never voting (not having voted in the past and planning not to vote in the future) in the countries of the former Soviet Union

| | Model 1<br>OR (95%CI) | Model 2<br>OR (95%CI) | Model 3<br>OR (95%CI) | Model 4<br>OR (95%CI) | Model 5<br>OR (95%CI) |
|---|---|---|---|---|---|
| Education | | | | | |
| High (N = 4327) | | | | | |
| Smoking status | | | | | |
| Never smoker | Ref. | Ref. | Ref. | Ref. | Ref. |
| Former smoker | 1.17 (0.86-1.59) | 1.26 (0.91-1.74) | 1.26 (0.91-1.74) | 1.21 (0.87-1.67) | 1.11 (0.80-1.54) |
| Current smoker | 1.26 (1.01-1.58)* | 1.33 (1.04-1.72)* | 1.34 (1.04-1.72)* | 1.28 (0.99-1.65) | 1.19 (0.91-1.54) |
| | | | | | |
| Mid (N = 9246) | | | | | |
| Smoking status | | | | | |
| Never smoker | Ref. | Ref. | Ref. | Ref. | Ref. |
| Former smoker | 1.07 (0.85-1.34) | 1.12 (0.88-1.43) | 1.12 (0.88-1.43) | 1.11 (0.87-1.42) | 1.08 (0.84-1.38) |
| Current smoker | 1.19 (1.04-1.37)* | 1.23 (1.04-1.46)* | 1.23 (1.04-1.47)* | 1.22 (1.02-1.45)* | 1.20 (1.00-1.43)* |
| | | | | | |
| Low (N = 2041) | | | | | |
| Smoking status | | | | | |
| Never smoker | Ref. | Ref. | Ref. | Ref. | Ref. |
| Former smoker | 0.43 (0.22-0.84)* | 0.59 (0.29-1.21) | 0.59 (0.29-1.21) | 0.59 (0.29-1.22) | 0.58 (0.28-1.20) |
| Current smoker | 1.29 (0.94-1.77) | 1.53 (1.01-2.33)* | 1.58 (1.04-2.40)* | 1.62 (1.05-2.51)* | 1.56 (1.01-2.41)* |

Model 1 examined the unadjusted association between smoking status and never voting; Model 2 additionally adjusted for sex, age, marital status, occupation, household finances and location; Model 3 was additionally adjusted for self-rated health; Model 4 was additionally adjusted for problem drinking; Model 5 was additionally adjusted for political distrust

All models were adjusted for country. Sample size varied between 4327 and 4342 for those in the lowest education group, 9246 and 9266 for those in the mid education group, and 2041 and 2055 for those in the high education group

OR: Odds ratio; CI: Confidence interval; Ref: Reference category

* $p<.05$

**Appendix 4** Household financial situation-specific associations between smoking status and never voting (not having voted in the past and planning not to vote in the future) in the countries of the former Soviet Union

| | Model 1<br>OR (95%CI) | Model 2<br>OR (95%CI) | Model 3<br>OR (95%CI) | Model 4<br>OR (95%CI) | Model 5<br>OR (95%CI) |
|---|---|---|---|---|---|
| Household finances | | | | | |
| Good/very good (N = 3410) | | | | | |
| Smoking status | | | | | |
| Never smoker | Ref. | Ref. | Ref. | Ref. | Ref. |
| Former smoker | 0.99 (0.68-1.44) | 1.02 (0.69-1.52) | 1.03 (0.69-1.52) | 1.03 (0.70-1.53) | 1.02 (0.68-1.52) |
| Current smoker | 1.33 (1.04-1.68)* | 1.41 (1.07-1.86)* | 1.41 (1.07-1.87)* | 1.43 (1.08-1.89)* | 1.38 (1.04-1.83)* |
| Average (N = 9042) | | | | | |
| Smoking status | | | | | |
| Never smoker | Ref. | Ref. | Ref. | Ref. | Ref. |
| Former smoker | 0.99 (0.79-1.25) | 1.10 (0.86-1.41) | 1.11 (0.87-1.41) | 1.09 (0.85-1.39) | 1.03 (0.80-1.32) |
| Current smoker | 1.13 (0.97-1.31) | 1.19 (0.99-1.42) | 1.20 (1.00-1.43) | 1.17 (0.97-1.40) | 1.12 (0.93-1.35) |
| Bad/very bad (N = 3157) | | | | | |
| Smoking status | | | | | |
| Never smoker | Ref. | Ref. | Ref. | Ref. | Ref. |
| Former smoker | 1.01 (0.69-1.49) | 1.26 (0.82-1.94) | 1.25 (0.81-1.93) | 1.22 (0.79-1.89) | 1.15 (0.74-1.78) |
| Current smoker | 1.30 (1.03-1.65)* | 1.51 (1.10-2.07)* | 1.51 (1.10-2.07)* | 1.44 (1.05-2.00)* | 1.40 (1.01-1.93)* |

Model 1 examined the unadjusted association between smoking status and never voting; Model 2 additionally adjusted for sex, age, education, marital status, occupation, and location; Model 3 was additionally adjusted for self-rated health; Model 4 was additionally adjusted for problem drinking; Model 5 was additionally adjusted for political distrust

All models were adjusted for country. Sample size varied between 3410 and 3426 for individuals whose household finances were good/very good, 9042 and 9066 for individuals whose household finances were average, 3157 and 3179 for individuals whose household finances were bad/very bad

OR: Odds ratio; CI: Confidence interval; Ref: Reference category

* p<.05

**Appendix 5** Self-rated health-specific associations between smoking status and never voting (not having voted in the past and planning not to vote in the future) in the countries of the former Soviet Union[†]

| | Model 1<br>OR (95%CI) | Model 2<br>OR (95%CI) | Model 3<br>OR (95%CI) | Model 4<br>OR (95%CI) |
|---|---|---|---|---|
| Self-rated health | | | | |
| Good/very good (N = 6144) | | | | |
| Smoking status | | | | |
| Never smoker | Ref. | Ref. | Ref. | Ref. |
| Former smoker | 0.93 (0.70-1.23) | 1.04 (0.78-1.40) | 1.00 (0.75-1.35) | 0.97 (0.72-1.31) |
| Current smoker | 1.07 (0.91-1.27) | 1.21 (0.99-1.47) | 1.16 (0.95-1.41) | 1.14 (0.93-1.39) |
| Fair (N = 6516) | | | | |
| Smoking status | | | | |
| Never smoker | Ref. | Ref. | Ref. | Ref. |
| Former smoker | 1.08 (0.82-1.42) | 1.17 (0.88-1.56) | 1.18 (0.88-1.57) | 1.11 (0.83-1.49) |
| Current smoker | 1.38 (1.16-1.64)** | 1.39 (1.12-1.72)* | 1.40 (1.13-1.75)* | 1.34 (1.08-1.67)* |
| Poor/very poor (N = 2958) | | | | |
| Smoking status | | | | |
| Never smoker | Ref. | Ref. | Ref. | Ref. |
| Former smoker | 0.90 (0.61-1.34) | 0.99 (0.62-1.59) | 0.98 (0.61-1.57) | 0.94 (0.58-1.51) |
| Current smoker | 1.16 (0.86-1.56) | 1.14 (0.77-1.68) | 1.12 (0.75-1.67) | 1.07 (0.72-1.61) |

[†]As the model was stratified by self-rated health, only 4 models were used in this analysis

Model 1 examined the unadjusted association between smoking status and never voting; Model 2 was additionally adjusted for sex, age, education, marital status, occupation, household finances and location; Model 3 was additionally adjusted for problem drinking; Model 4 was additionally adjusted for political distrust

All models were adjusted for country. Sample sizes varied between 6144 and 6159 for those with good/very good self-rated health, 6516 and 6529 for those with fair self-rated health and 2958 and 2973 for those with bad/very bad self-rated health

OR: Odds ratio; CI: Confidence interval; Ref: Reference category

** p<.001, * p<.01
